# Bidisperse Magnetic Particles Coated with Gelatin and Graphite Oxide: Magnetorheology, Dispersion Stability, and the Nanoparticle-Enhancing Effect

**DOI:** 10.3390/nano8090714

**Published:** 2018-09-11

**Authors:** Yu Fu, Jianjun Yao, Honghao Zhao, Gang Zhao, Zhenshuai Wan, Ying Qiu

**Affiliations:** 1 College of Mechanical and Electrical Engineering, Harbin Engineering University, Harbin 150001, China; 18345032884@163.com (Y.F.); travisyao@126.com (J.Y.); zhaogang@hrbeu.edu.cn (G.Z.); 18242311944@163.com (Z.W.); qiuying@hrbeu.edu.cn (Y.Q.); 2 Department of Decision Sciences, School of Business, Macau University of Science and Technology, Macau 999078, China

**Keywords:** magnetorheological fluids, bidisperse magnetic particles, gelatin, graphite oxide, carbonyl iron, nanoparticles Fe_3_O_4_

## Abstract

The magnetorheology and dispersion stability of bidisperse magnetic particles (BMP)-based magnetorheological (MR) fluids were improved by applying a novel functional coating composed of gelatin and graphite oxide (GO) to the surfaces of the micron-sized carbonyl iron (CI) and nanoparticles Fe_3_O_4_. Gelatin acted as a grafting agent to reduce the aggregation and sedimentation of CI particles and prevent nanoparticles Fe_3_O_4_ from oxidation. In addition, a dense GO network on the surface of gelatin-coated BMP was synthesized by self-assembly to possess a better MR performance and redispersibility. The rheological properties of MR fluids containing dual-coated BMP were measured by a rotational rheometer under the presence of magnetic field and their dispersion stability was examined through sedimentation tests. The results showed that CI@Fe_3_O_4_@Gelatin@GO (CI@Fe_3_O_4_@G@GO) particles possessed enhanced MR properties and dispersion stability. In addition, the nanoparticle-enhancing effects on the dispersion stability of BMP-based MR fluids were investigated using Monte Carlo simulations.

## 1. Introduction

Magnetorheological (MR) fluid, the suspension of soft magnetic particles dispersed in a nonmagnetic liquid medium, exhibiting a rapid and reversible change from fluid-like to solid-like state when subjected to the magnetic fields, has been considered to be one of the most promising intelligent materials [1,2,3,4,5,6]. MR fluids show a superior rheological behavior under the presence of magnetic field and have been widely used in many engineering applications including dampers, torque transducers, clutches, and actuators [7,8,9,10,11]. However, MR fluids still require necessary improvement in their rheological properties and dispersion stability due to the inherent sedimentation behavior of micron-sized magnetic particles.

Recently, it was reported that the filling with nanoparticles was an effective method to reduce the sedimentation of larger particles in MR fluids [12,13,14,15,16,17,18,19,20]. Leong et al. [21] prepared bidisperse magnetic particles (BMP) composed of *γ*-Fe_2_O_3_ nanoparticles with an average size of 9 nm and carbonyl iron (CI) particles to investigate the rheological properties and sedimentation rate of the MR fluids. Wereley et al. [22] attemped to find an optimal composition of micron-sized and nanosized Fe particles to provide the best combination of high yield stress and low sedimentation rate for bidisperse MR fluids. Patel [23] then studied the mechanism of structure formation in magnetic nanofluid based MR fluids and indicated that nanofluid-based MR fluids possessed better stability compared with the commercially available MR fluids. When the external magnetic field is present, the micron-sized particles become polarized and gathered in chains or clusters owing to dipole–dipole interaction [24]. Simultaneously, nanoparticles attached at the end of CI chains and filled into the microcavities formed by CI particles can influence the interactions among CI particles and reduce the aggregation of larger particles for BMP-based MR fluids.

Nevertheless, the nanoparticles Fe_3_O_4_ are susceptible to be oxidized at elevated temperatures and transformed into *α*-Fe_2_O_3_ with better thermal stability losing their magnetic properties. Thus, the core-shell structures composed of magnetic particle and polymer have been prepared, in which the various polymers including polystyrene (PS), polyaniline (PANI), and silica were introduced to improve the stability of magnetic particles [25,26,27,28]. Unfortunately, as a result of the coating of non-magnetic polymer, the magnetic interactions between magnetic particles are diminished, which inevitably affects the MR properties.

Consequently, we constructed a dense graphite oxide (GO) nest on the surface of micron-sized CI and nanosized Fe_3_O_4_ particles using gelatin as a grafting agent, which is also called CI@Fe_3_O_4_@Gelatin@GO (CI@Fe_3_O_4_@G@GO) particles. Gelatin is obtained by alkali denaturation of collagen. The alkaline denaturation process targets the –NH_2_ groups of asparagines and glutamine, and hydrolyzes them into –COOH, hence converting these residues into aspartate and glutamate. This results in the alkaline-processed gelatin possessing a high proportion of –COOH thereby rendering it negatively charged and lowering its isoelectric point. Subsequently, the core-shell structure morphology and composition analysis of CI@Gelatin (CI@G), CI@Fe_3_O_4_@Gelatin (CI@Fe_3_O_4_@G), and CI@Fe_3_O_4_@G@GO particles were confirmed from scanning electron microscope (SEM), X-ray energy dispersive (XRD), and Fourier transform infrared (FT-IR) spectra. In addition, MR properties (shear stress, storage modulus, and yield stress) and dispersion stability for modified particle-based MR fluids were examined and compared to those of pure CI particles. Additionally, the nanoparticle-enhancing effects on the dispersion stability for BMP-based MR fluids were illustrated.

## 2. Materials and Methods 

### 2.1. Synthesis of Gelatin Coated CI@Fe_3_O_4_ (CI@Fe_3_O_4_@G) Particles

At first, the grafting agent (2.5 g), gelatin, was dissolved in 25 mL of deionized water and heated to 55 °C for 30 min. Four grams of micron CI particles (Fe% > 98%, average diameter = 3.5 μm, density = 7.9 g/cm^3^), nanoscale Fe_3_O_4_ particles (average diameter = 20 nm) and 0.2 g of sodium chloride were successively added to the mixture solution. The nanoscale Fe_3_O_4_ particles were prepared by the classical co-precipitation method [29]. The sulfate solution containing Fe^3+^ and Fe^2+^ was added into an alkaline solution, which was continuously stirred for 30 min at pH 10.5 to allow nanocrystallites growing in size. The nanocrystallites were magnetically separated and washed several times with deionized water to remove water-soluble impurities, and dried in a vacuum oven at 55 °C for 12 h. Herein, the sodium chloride in the solution played a role as a stabilizing agent to maintain the balance of interaction force between CI and nanoscale Fe_3_O_4_ particles.

The resulting reaction mixture was treated with ultrasonic oscillation for 6 h, so the surface-modified CI and Fe_3_O_4_ (CI@Fe_3_O_4_@G) particles were obtained. The products were then separated by a strong magnet and washed with deionized water in order to remove excess gelatin. The appropriate mass fraction of nanoparticles Fe_3_O_4_ in the preparation process is given in the next section.

### 2.2. Fabrication of the GO-Nest Wrapped CI@Fe_3_O_4_@G (CI@Fe_3_O_4_@G@GO) Particles

GO was prapared using a modified Hummer’s process [30,31]. The graphite, NaNO_3_, H_2_SO_4_, MnSO_4_·H_2_O, KMnO_4_, H_2_O_2_, and FeC_2_O_4_·2H_2_O were directly employed without further treatment. First, 2 g of graphite and 1 g of NaNO_3_ were placed into a 250 mL flask, followed by the dropwise addition of 25 mL of H_2_SO_4_ while stirring in an ice-bath. Three grams of KMnO_4_ were then added to the mixture, which was continuously stirred to the room temperature and diluted with 100 mL of deionized water, followed by the addition of 30% H_2_O_2_ solution to remove excess KMnO_4_. The final GO materials were obtained after centrifuging and washing (with deionized water), and then dried at 70 °C for 24 h.

**Figure 1:**
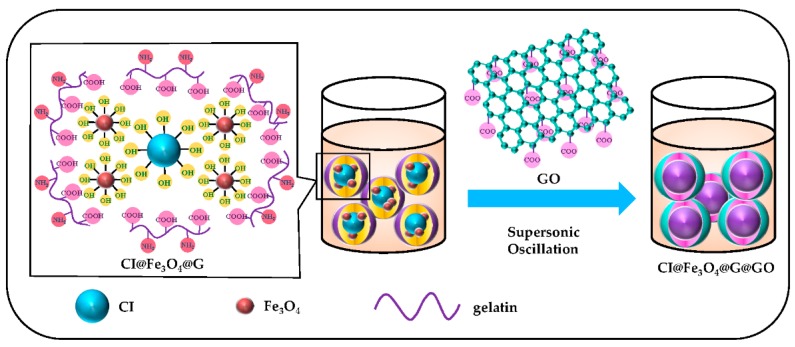
Schematic diagram of the preparation process for CI@Fe_3_O_4_@G@GO magnetic particles.

Secondly, 2 g of GO was added to the previously synthesized solution containing CI@Fe_3_O_4_@G particles and deionized water. The mixture was stirred for 1 h at 55 °C. After ultrasonic oscillation for 10 h, the surface of CI@Fe_3_O_4_@G particles were wrapped with GO nest. Furthermore, the CI@Fe_3_O_4_@G@GO paticles were separated by a permanent magnet and washed with deionized water until the supernatant liquid became colorless. A schematic diagram of the CI@Fe_3_O_4_@G@GO particles steps is provided in Figure 1.

### 2.3. Characterization Methods

In this work, SEM images of the particles morphologies were taken on a JSM-7500F electron microscope (JEOL Ltd., Co., Tokyo, Japan) at an acceleration voltage of 5.0 kV. X-ray EDS was also performed using an attached EDAX (couple with JSM-7500F) spectrometer (JEOL Ltd., Co., Tokyo, Japan) for the elemental analysis.

FT-IR spectra of the dried CI@G, CI@Fe_3_O_4_@G, and CI@Fe_3_O_4_@G@GO particles was obtained on an FT-IR iS50 spectrometer (Thermo Fisher Scientific Co., Waltham, MA, USA) in the range of 4000–500 cm^−1^.

XRD for the dried magnetic particles of pure CI, pure Fe_3_O_4_, CI@Fe_3_O_4_@G, and CI@Fe_3_O_4_@G@GO was recorded on a TTR-III X-ray diffractor (Rigaku, Tokey, Japan) using a Cu Kα tube and an Ni filter in the 2*θ* range of 20–90° with an interval of 0.02°.

Magnetorheological behaviors of the modified particle-based MR fluids were investigated with a rotational magneto-rheometer MCR-302 MRD (Anton Paar, Graz, Austria) at room temperature. In order to prepare the MR fluid, synthesized CI@G, CI@Fe_3_O_4_@G, and CI@Fe_3_O_4_@G@GO as well as pure CI particles were dispersed in the mixture of polyolefins synthetic oil, which were stirred for 40 min at 50 °C, respectively. In addition, the dispersion stability of MR fluid was tested by qualitative observation of sedimentation at room temperature. In this method, the sedimentation ratio *β* can be defined as

(1) $$\beta =\frac{H-h}{H}\times 100\%\;$$

where *H* and *h* represent the initial and residual heights of MR fluids, respectively.

## 3. Results and Discussion

### 3.1. Nanoparticle-Enhancing Effect

**Figure 2:**
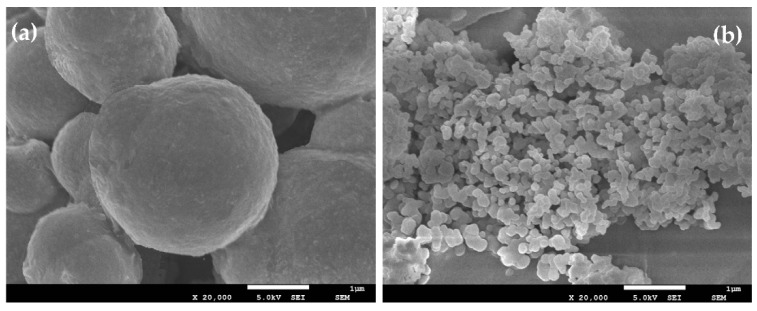
SEM images of microstructure morphology: (**a**) pure micron-sized CI particles; (**b**) pure nanoparticles Fe_3_O_4_.

For magneto-controllable MR fluids, it is widely recognized that the dispersion stability depended on their interior microstructures. Figure 2 shows the SEM images of microstructure morphology for micron-sized CI and nanoscale Fe_3_O_4_ particles, respectively, which exhibits a very smooth surface with a regular shape. However, we lack a complete explanation of the formation mechanism for BMP with different sizes, magnetic properties, and mechanical performance. In this section, the microstructure-based machanism of the nanoparticle-enhancing effect was illustrated in BMP-based MR fluids.

Generally, in the BMP-based MR fluids, due to dipolar interactions, Fe_3_O_4_ particles are attached to CI chains or filled into the microcavities formed by larger particles, which significantly influences the interactions among CI particles. Magnetic force can be described by the interaction of induced dipoles. The magnetic moment of a magnetic particle is given by

(2) $$m=\frac{4}{3}\pi R^{3}\chi H\;$$

*H* is the applied magnetic strength, and *R* and *χ* are the radius and magnetic susceptibility of the particle. The magnetostatic energy *E_H_* can be expressed as

(3) $$E_{H}=-m\times H\;$$

When a particle marked as *i* is magnetized as a dipole, it will generate a magnetic field in the surroundings:

(4) $$H_{m}{}_{{}_{i}}=\frac{\mu_{0}}{4\pi r_{0}^{3}}\left[\frac{3\left(m_{i}\times r_{0}\right)\times r_{0}}{r^{2}}-m_{i}\right]\;$$

The magnetic potential energy *E_mimj_* between dipoles *i* and *j* can be expressed as

(5) $$E_{m}{{}_{{}_{i}}}_{m}{}_{{}_{j}}=\frac{\mu_{0}}{4\pi r_{ij}^{3}}\left[\frac{3\left(m_{i}\times r_{0}\right)\left(m_{j}\times r_{0}\right)}{r^{2}}+m_{i}\times m_{j}\right]\;$$

where *r_ij_* is the position vector from dipole *j* to dipole *i*, and *r*_0_ is a unit vector of *r_ij_*. The interparticle magnetic force between dipoles *i* and *j* is obtained as

(6) $$F_{m}{{}_{{}_{i}}}_{m}{}_{{}_{j}}=\frac{3\mu_{0}}{4\pi r_{ij}^{4}}\left[-3\left(m_{i}\times r_{0}\right)\left(m_{j}\times r_{0}\right)+m_{i}\times m_{j}\right]\;$$

Therefore, the sum of magnetic forces exerted by the surrounding magnetic particles can be given by

(7) $$F_{m}=\sum_{j\neq i}\frac{3\mu_{0}}{4\pi r_{ij}^{4}}\left[-3\left(m_{i}\times r_{0}\right)\left(m_{j}\times r_{0}\right)+m_{i}\times m_{j}\right]\;$$

The van der Waals force for dissimilar particles of radius *R*_1_ and *R*_2_ can be calculated by Equations (8) and (9) [23]:

(8) $$F_{v}=-\frac{A_{212}}{6h}\frac{R_{1}R_{2}}{R_{1}+R_{2}}\;$$

(9) $$A_{212}=\left(A_{1}^{\frac{1}{2}}-A_{2}^{\frac{1}{2}}\right)\;$$

*A* is the Hamaker constant. CI and Fe_3_O_4_ particles are denoted by subscripts 1 and 2, respectively, The subscript 212 represents the orderly distribution of CI and Fe_3_O_4_ particles in the MR fluids. Herein, *A*_1_ = 7.24 × 10^−20^ J and *A*_2_ = 1.0 × 10^−19^ J. *A*_212_ is an effective Hamaker constant and taken as 2.22 × 10^−21^ J. In addition, it was found that the magnitude of van der Waals force generally increased with the decrease of intermolecular distance *h*.

In addition, Brownian motion force has an important effect on preventing the aggregation of micron-sized particles, which can be obtained by Equation (10) [32]:

(10) $$F_{B}=\zeta \sqrt{\frac{12\pi R_{a}\mu k_{B}T}{\Delta t}}\;$$

where *ζ* is a random number of Gaussian distribution, *R*_a_ is the mean radius of BMP, *k*_B_ is Boltzmann constant, *μ* is dynamic viscosity, *T* is thermodynamic temperature, and Δ*t* is time step. These forces are major factors affecting the dispersion stability of MR fluids.

In this work, nanoparticles Fe_3_O_4_ were applied as a form of hybrids with CI particles to prepare MR fluids. For the BMP-based MR fluids, the mass fraction of nanoparticles is denoted as *w*. In order to understand the microstructure-based mechanism of the nanoparticle-enhancing effect, the microstructure in a three-dimensional (3D) case is studied by Monte Carlo simulations [33].

For magnetic particle *i*, the initial position $\phi_{i}^{t}$ is defined as follows:

(11) $$\phi_{i}^{t}=\left(x_{i}^{t},y_{i}^{t},z_{i}^{t}\right)\;$$

After initialization, the corresponding positions are subjected to multi-interation in the movement process:

(12) $$x_{i}^{t+1}=x_{i}^{t}+\left(R_{1}-0.5\right)\Delta \;$$

(13) $$y_{i}^{t+1}=y_{i}^{t}+\left(R_{2}-0.5\right)\Delta \;$$

(14) $$z_{i}^{t+1}=z_{i}^{t}+\left(R_{3}-0.5\right)\Delta \;$$

where *R* is a random number, and Δ is the maximum displacement allowed. The optimal value can be obtained by [34]

(15) $$\left\{ \begin{array}{l} \beta_{t+1}=\lambda \beta_{t}+\theta \left(\mathrm{mod}M\right)\;t=0,1,\ldots ,n\\ R=\frac{\beta_{t+1}}{M} \end{array}\right.\;$$

where *λ*, *θ*, and *M* represent the multiplier, increment, and modulus of the Monte Carlo, respectively. Therefore, the position Φ*^t^* of magnetic particle group is determined as

(16) $$\Phi^{t}=\left(\phi_{1}^{t},\phi_{2}^{t},\ldots ,\phi_{n}^{t}\right)\;$$

The random probability *P_i_* of position updating depends on the change of particle energy (Δ*Ε* = $E_{+1I}^{t}$ − $E_{I}^{t}$), and their relationship can be given as

(17) $$P_{i}=\left\{ \begin{array}{ll} \exp\left(-\frac{\Delta E}{kT}\right)\; & \Delta E>0\\ 1 & \Delta E<0 \end{array}\right.$$

In MR fluids, the energy in magnetic particles includes gravity gradient energy, kinetic energy, magnetostatic energy, magnetic potential energy, and van der Waals force energy. However, the gravity gradient energy and kinetic energy have little change during the movement process, and van der Waals force energy can be neglected. Thereby, the change in energy is mainly influenced by magnetostatic energy and magnetic potential energy, which can be obtained by Equations (3) and (5), respectively.

**Figure 3:**
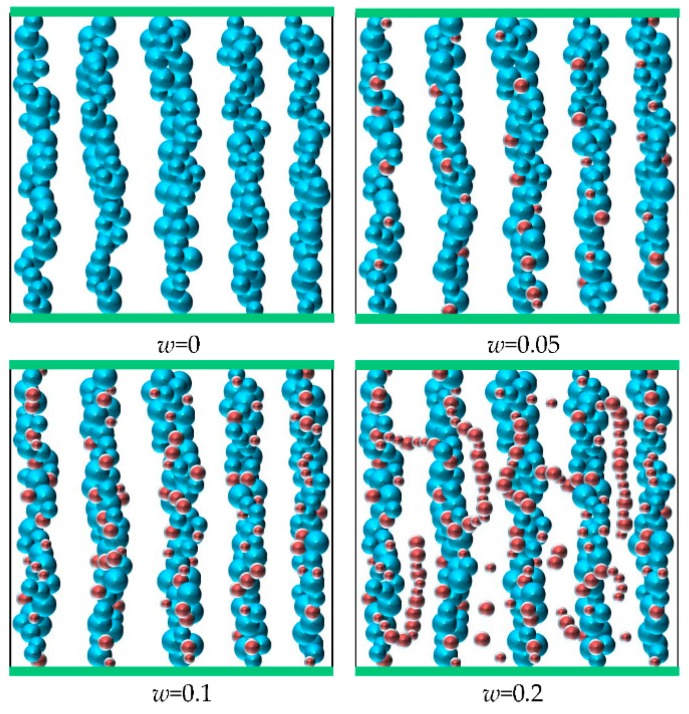
The magneto-induced microstructures with *w* = 0, 0.05, 0.1, and 0.2, respectively. The larger blue balls denote the CI particles and the red balls denote the Fe_3_O_4_ particles.

In this simulation, a cubic cell with edge length 50 μm is considered for the 3D case. The microstructure diagram with different mass fraction of nanoparticles are presented in Figure 3. When *w* = 0, the CI particles form chain-like structures along the direction of the external magnetic field. However, larger soft magnetic materials are rather easy to be magnetized and aggregated, thus resulting in rapid and serious sedimentation. For the condition of *w* = 0.05, namely there is a small number of nanoparticles in the total particles, the main microstructures are also the chain-like structures formed by CI particles. Meanwhile, the nanoparticles are mainly separated from each other and attached to the CI chains or filled into the microcavities of CI particles. When *w* increases to 0.1, some nanoparticles form short chain-like structures filling the interspace among CI chains. Thus, the dipolar interactions and van der Waals forces between magnetic particles are enhanced, which improves the sedimentation behaviors of MR fluids. Increasing *w* to 0.2, nanoparticles play a considerable role contributing to the whole structure. The nanoparticles are aggregated to form long chain-like structures and filled around the CI chains. However, the excess nanoparticles are dispersed in the carrier liquid, resulting in the augmentation of zero-field viscosity and decrease of magnetic properties for MR fluids.

Therefore, the microstructure mechanism reveals that magneto-induced short chain-like stuctures formed by nanoparticles contribute to the dispersion-enhancing effect. When the mass fraction of nanoparticles is about 10%, the BMP-based MR fluid possesses better dispersion stability.

### 3.2. Particle Morphology and Chemical Composition

**Figure 4:**
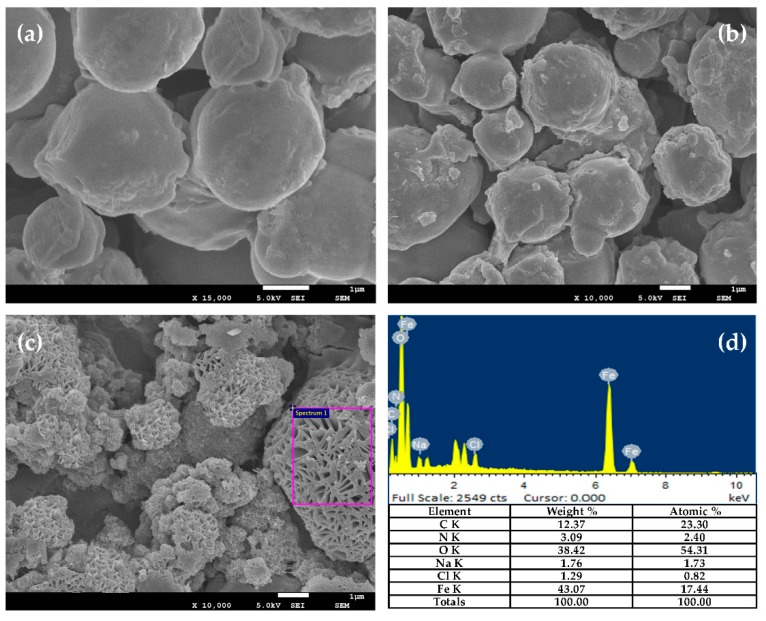
SEM images of (**a**) CI@G particles, (**b**) CI@Fe_3_O_4_@G particles, (**c**) CI@Fe_3_O_4_@G@GO particles, and (**d**) EDS analysis of CI@Fe_3_O_4_@G@GO particles.

Figure 4 displays the SEM images of modified particle morphology. The spherical CI@G and CI@Fe_3_O_4_@G particles (Figure 4a,b) exhibits a clear core-shell structure with gelatin on the surface, and an obvious difference on the surface of CI@G and CI@Fe_3_O_4_@G is observed due to the filling of nanoparticles. Figure 4c,d show the surface morphology and EDS analysis of CI@Fe_3_O_4_@G@GO particles, respectively. It can be found that modification with GO introduced chemical functional groups to the CI and Fe_3_O_4_ surfaces, which resulted in quite rough surfaces, and the interspace among the particles was reduced due to the wrapping of GO nest. Additionally, typical EDS spectra indicated strong intensities of organic elements carbon, nitrogen, and oxygen, originating from gelatin and GO. Considering that the CI@Fe_3_O_4_@G@GO particles have been washed with deionized water to remove excess GO and gelatin, we confirmed the successful coating of BMP with gelatin and GO.

**Figure 5:**
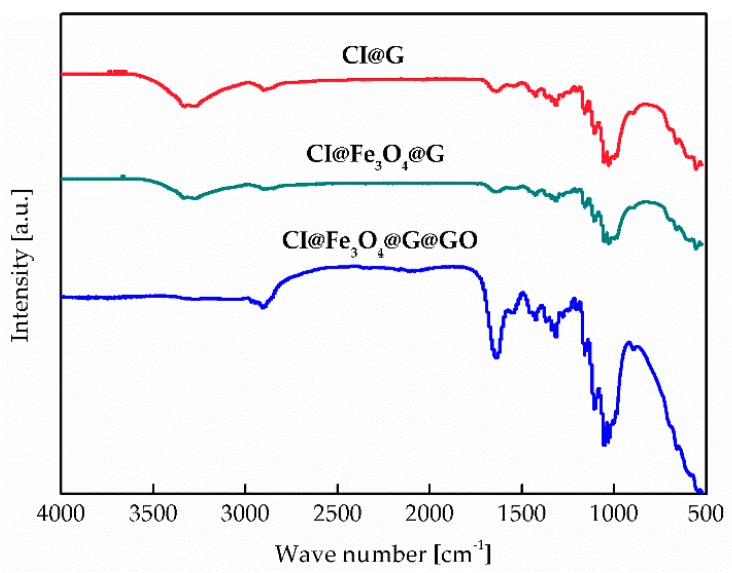
FT-IR spectra of CI@G, CI@Fe_3_O_4_@G, and CI@Fe_3_O_4_@G@GO particles.

The functional groups in CI@G, CI@Fe_3_O_4_@G, and CI@Fe_3_O_4_@G@GO particles were investigated by FT-IR spectroscopy (Figure 5). The U-shaped polypeptide chain of gelatin exhibits an extensional planar structure and its –C=O and –C–N groups are perpendicular to the chain axis. In the spectrum of CI@G, CI@Fe_3_O_4_@G, and CI@Fe_3_O_4_@G, there were several common absorption peaks: C=O (1640 cm^−1^), C–H (1247 cm^−1^), C–N (1053 cm^−1^), and Fe–O (556 cm^−1^). This confirmed the presence of gelatin functional groups on the surface of BMP. Furthermore, the spectra of CI@G and CI@Fe_3_O_4_@G particles showed double absorption peaks of primary amine located at 3332 cm^−1^ and 3429 cm^−1^, which indicated the existence of the –NH_2_ groups. The characteristic C=O peak in the spectra of CI@Fe_3_O_4_@G@GO was strengthened compared to the other particles on account of the increase in quantity. In order to prove the role of gelatin as a grafting agent, an additional experiment without adding gelatin was also made. Unfortunately, there was not any wrapped GO layer on the surface of BMP, which indicates that the gelatin plays a crucial role wrapping BMP with GO nests. We assumed that, in an aqueous system, CI and Fe_3_O_4_ particles were coordinated to water molecules that shared their electron pairs with the iron atoms, and resulted in a surface covered by –OH groups. Furthermore, –COOH groups in gelatin had a better adsorption on the –OH groups due to esterification reaction. After modifying the CI@Fe_3_O_4_@G particles in a bath, the epoxide group on the surface of GO reacted with –NH_2_ in the gelatin. Therefore, a mass of the GO densely covered the surface, which led to the disappearance of double absorption peaks of –NH_2_ in the spectra of CI@Fe_3_O_4_@G@GO particles.

**Figure 6:**
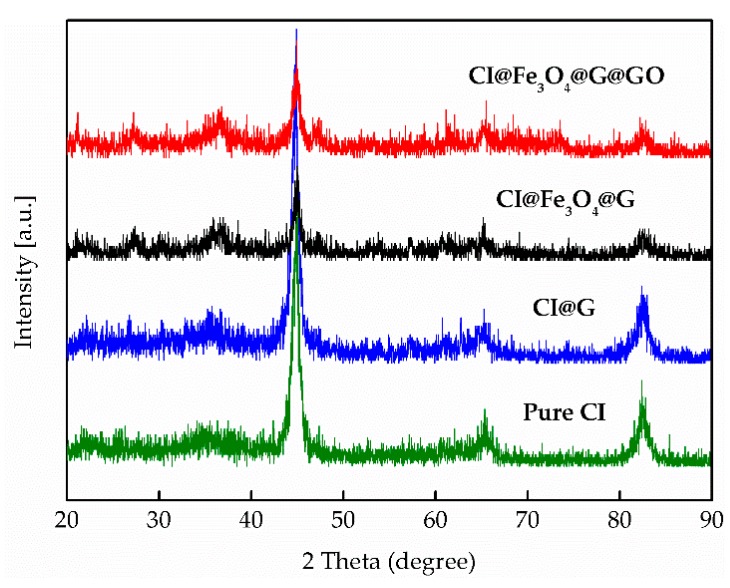
XRD spectra of pure CI, CI@G, CI@Fe_3_O_4_@G, and CI@Fe_3_O_4_@G@GO particles.

XRD was employed to investigate the crystalline structures of modified particles. The characteristic diffractions of the pure CI, CI@G, CI@Fe_3_O_4_@G, and CI@Fe_3_O_4_@G@GO particles were shown in Figure 6. The characteristic peaks of CI@G, CI@Fe_3_O_4_@G, and CI@Fe_3_O_4_@G@GO particles were the same as the unmodified pure CI particles, which showed that the contents of GO did not affect the inherent crystalline characteristics of CI particles.

### 3.3. MR Behavior and Dispersion Stability

**Figure 7:**
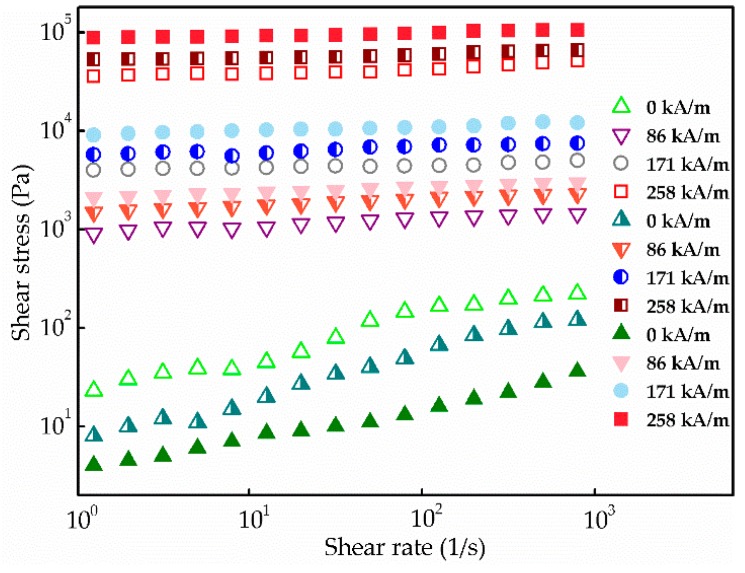
Shear stress curves for MR fluids based on CI@G (open symbol), CI@Fe_3_O_4_@G (semiclosed symbol), and CI@Fe_3_O_4_@G@GO particles (closed symbol) under various magnetic field strengths.

The MR fluid behaviors were characterized at different magnetic fields ranging from 0 to 258 kA/m under a rotational test. For a typical MR behavior, shear stress was measured on a log–log scale applying different external magnetic fields. As shown in Figure 7, the shear stress of MR fluids containing CI@G, CI@Fe_3_O_4_@G, and CI@Fe_3_O_4_@G@GO particles increased with the increasing shear rate. These steady behaviors were induced based on a solid-like chain formation in which the magnetic moments of the particles were parallel to the magnetic field direction. When the magnetic field was present, the MR fluids with CI@Fe_3_O_4_@G@GO particles displayed relatively higher shear stress than CI@G and CI@Fe_3_O_4_@G particles due to the increased friction caused by the rough coating of GO nest. As expected, the shear stress achieved highly depended on the applied magnetic fields, and all shear stress curves represented a wide plateau range over the whole region of shear rate due to the strong dipole–dipole interactions among the adjacent magnetic particles. On the other hand, when the magnetic field was absent, the disorganized Brownian motion performed by BMP reduced the flocculation between the magnetic particles and led to the decrease on the shear stress of CI@Fe_3_O_4_@G and CI@Fe_3_O_4_@G@GO particles.

**Figure 8:**
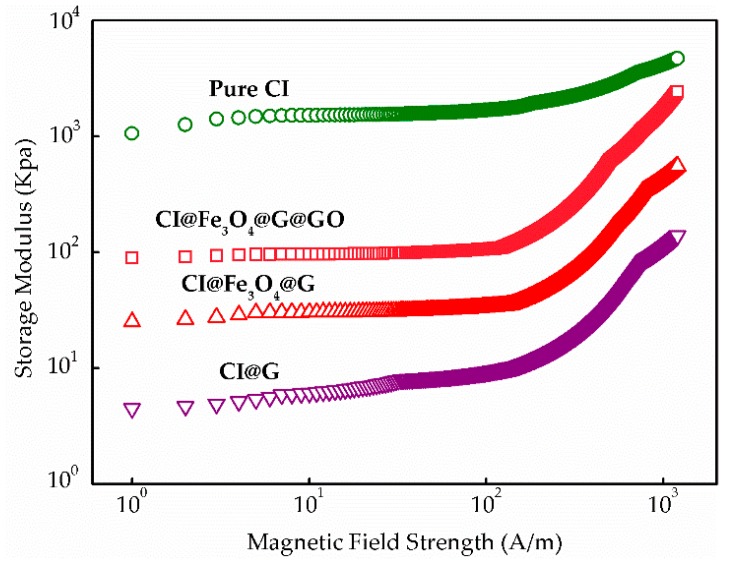
Magnetic field response properties for MR fluids based on pure CI, CI@G, CI@Fe_3_O_4_@G and CI@Fe_3_O_4_@G@GO particles.

Figure 8 showed the relationship between storage modulus and magnetic field strengths applied for MR fluids. It is found that gelatin-coated layers of particles reduced magnetic response time compared with that of the pure CI particles, in which the response time was evaluated by the transition point where the slope of storage modulus significantly chanced. However, the response velocity of CI@Fe_3_O_4_@G@GO particles became slightly faster than that of gelatin-coated particles. As the magnetic field increased, the storage modulus of CI@G, CI@Fe_3_O_4_@G, and CI@Fe_3_O_4_@G@GO particles rapidly increased compared with the slope of the pure CI. It may be due to the fact that applied magnetic fields were disturbed by the gelatin-coated layer on particles. In addition, it was observed that the storage modulus of CI@Fe_3_O_4_@G@GO was always higher than that of CI@G and CI@Fe_3_O_4_@G over the entire magnetic field range. This suggests that the MR fluids containing CI@Fe_3_O_4_@G@GO possessed a solid-like behavior, indicating more remarkable elastic properties.

**Figure 9:**
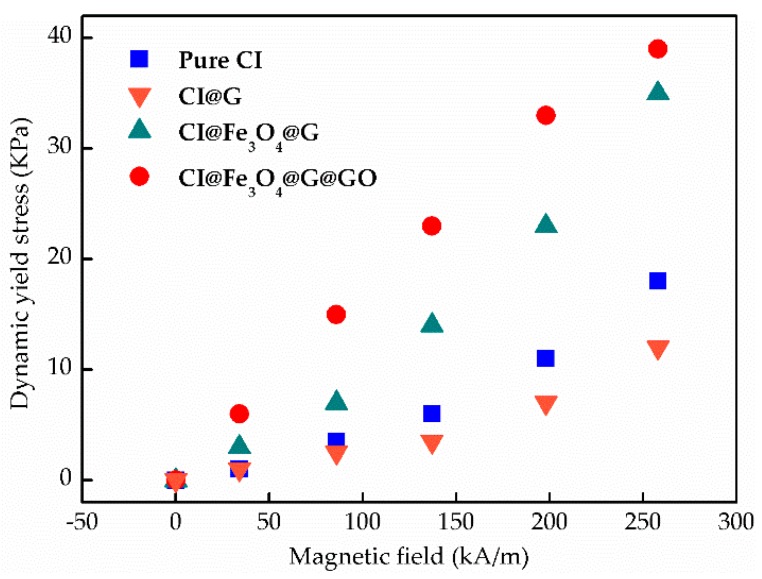
Plots of dynamic yield stress vs. magnetic field of MR fluids based on pure CI, CI@G, CI@Fe_3_O_4_@G and CI@Fe_3_O_4_@G@GO particles.

In addition, the dynamic yield stress as a function of magnetic fields was shown in Figure 9. The dynamic yield stress of all particles indicated an increasing tendency with increasing applied magnetic fields. CI@Fe_3_O_4_@G@GO particles showed the highest values of yield stress. On the other hand, the CI@G particles displayed slightly weaker dynamic yield stresses than pure CI particles for all magnetic field strengths applied.

**Figure 10:**
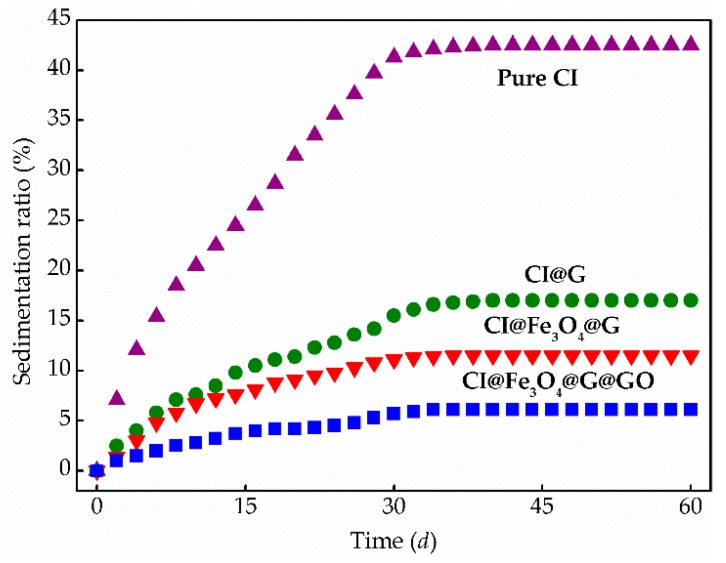
Sedimentation profile recorded as a function of time for MR fluids based on pure CI, CI@G, CI@Fe_3_O_4_@G, and CI@Fe_3_O_4_@G@GO particles.

Furthermore, the sedimentation observation of MR fluids containing pure CI, CI@G, CI@Fe_3_O_4_@G, and CI@Fe_3_O_4_@G@GO particles was examined. Figure 10 showed the recorded sedimentation ratio as a function of time. Apparently, compared with pure CI particles, CI@G, CI@Fe_3_O_4_@G, and CI@Fe_3_O_4_@G@GO particles exhibited slow sedimentation velocity during the initial 34 days and then tended to become steady at 17.2%, 10.3%, and 6.58%. The lower the sedimentation ratio is, the better the dispersion stability is. It is obvious that dual-coated layers and nanoparticles significantly affect the dispersion stability of MR fluids, and the sedimentation ratio for CI@Fe_3_O_4_@G@GO particles is lower than 7.00%, which is superior to many MR fluids in the literature [35,36].

In conclusion, under the effect of magnetic field, Fe_3_O_4_ particles attached at the end of CI chains and filled into the microcavities formed by larger particles influence the interactions among CI particles. However, the nanoparticles Fe_3_O_4_ are susceptible to be oxidized at elevated temperatures losing their magnetic properties. Consequently, as a grafting agent, gelatin layers can restrict the oxidation of nanoparticles and reduce the attraction among the adjacent particles due to the increased lubrication of particle surfaces to improve the dispersion stability of magnetic particles. Nevertheless, as a result of the coating of the non-magnetic layer, the magnetic interactions between magnetic particles are diminished, which inevitably leads to the decrease of MR properties.

After being wrapped with GO nest, considerably coarse surfaces may be produced. When the external magnetic field is present, these coarse particles will inevitably suffer interaction, hence absorbed GO nest can produce flocculation by bridging the gap between the adjacent CI particles, which enhances the MR properties. A homogeneous redispersion of the MR fluid containing CI@Fe_3_O_4_@G@GO particles could be obtained easily by momentary mild shaking and allowing sufficient time for the particles to settle. Therefore, compared with other particles, CI@Fe_3_O_4_@G@GO particles possess enhanced dispersion stability and easy redispersion.

## 4. Conclusions

In this work, dual-coated BMP with a dense GO network was fabricated using gelatin as the grafting agent. The core-shell structure morphology and composition analysis were confirmed from SEM, FT-IR, and XRD spectra. Rheological properties of MR fluids containing dual-coated BMP were measured by a rotational rheometer under the presence of magnetic field and their dispersion stability was examined through sedimentation tests. The CI@Fe_3_O_4_@G@GO particles indicate better MR properties than that of pure CI, CI@G, and CI@Fe_3_O_4_@G particles. Simultaneously, MR fluids containing CI@Fe_3_O_4_@G@GO particles exhibit better dispersion stability and redispersibility. The proposed MR fluids containing CI@Fe_3_O_4_@G@GO particles may serve for future research in many engineering applications. In addition, the nanoparticle-enhancing effects on the dispersion stability of BMP-based MR fluids were studied using Monte Carlo simulations. When the mass fraction of nanoparticles is about 10%, some nanoparticles will form short chain-like structures or become attached to the end of CI chains, filling the interspace among CI chains, strongly increasing the dipolar interactions and van der Waals forces between BMP, thus enhancing its dispersion stability.
